# Supplementary material for: Association between age people started working and missing teeth in an elderly population in Ecuador: Evidence from a cross-sectional study
Source: PLoS One. 2023 Nov 13;18(11):e0293635. doi: 10.1371/journal.pone.0293635 (PMC10642810; doi:10.1371/journal.pone.0293635)
Supplement: S1 Table — showcases a non-weighted sensitivity analysis of predictor and control variables related to the outcome of "missing more than 4 teeth" across two models: a bivariate model (Model 1) and a model incorporating all controls (Model 2). The table provides odds ratios, standard errors (SE), and 95% confidence intervals (CI) for various demographic, health, and lifestyle factors, including age, sex, educational level, ethnicity, marital status, health conditions, smoking status, childhood socioeconomic situation, and insurance type, with data drawn from a sample of 3,899 individuals. (DOCX) [file pone.0293635.s001.docx]

**S1 Table.** Non-weighted sensitivity analysis of predictor and control variables.

| **Variables** | **Model 1: Missing more than 4 teeth** | **Model 2: Missing more than 4 teeth** |
| --- | --- | --- |
|  |  |  |
| **Age when first started working = 1, 5-9** | 1.439** | 1.551** |
|  | (SE 0.251) | (SE 0.296) |
|  | [95% CI 1.022- 2.025] | [95% CI 1.068 - 2.254] |
| **Age when first started working = 2, 10-19** | 1.164 | 1.298* |
|  | (SE 0.141) | (SE 0.176) |
|  | [95% CI 0.918-1.477] | [95% CI 0.995-1.693] |
| **Age when first started working = 4, 60-80** | 2.666 | 1.739 |
|  | (SE 2.757) | (SE 1.831) |
|  | [95% CI 0.351-20.239] | [95% CI 0.221-13.697] |
| **Sex = Male** |  | 0.434*** |
|  |  | (SE 0.0581) |
|  |  | [95% CI 0.334 - 0.564] |
| **Age = 2, 66-70** |  | 1.752*** |
|  |  | (SE 0.224) |
|  |  | [95% CI 1.363 - 2.252] |
| **Age = 3, 71-75** |  | 2.751*** |
|  |  | (SE 0.432) |
|  |  | [95% CI 2.022 - 3.743] |
| **Age = 4, 76-80** |  | 4.359*** |
|  |  | (SE 0.907) |
|  |  | [95% CI 2.899 - 6.553] |
| **Age = 5, 81+** |  | 6.761*** |
|  |  | (SE 1.678) |
|  |  | [95% CI 4.157 - 10.997] |
| **Educational level = 1, Primary** |  | 0.918 |
|  |  | (SE 0.133) |
|  |  | [95% CI 0.692-1.219] |
| **Educational level = 2, Secondary** |  | 0.715* |
|  |  | (SE 0.145) |
|  |  | [95% CI 0.481-1.063] |
| **Educational level = 3, Postsecondary** |  | 0.435*** |
|  |  | (SE 0.105) |
|  |  | [95% CI 0.271-0.698] |
| **Ethnic self-report = 2, Black** |  | 0.777 |
|  |  | (SE 0.206) |
|  |  | [95% CI 0.463 - 1.305] |
| **Ethnic self-report = 3, White** |  | 0.964 |
|  |  | (SE 0.154) |
|  |  | [95% CI 0.705 - 1.320] |
| **Ethnic self-report = 4, Indigenous** |  | 0.790 |
|  |  | (SE 0.136) |
|  |  | [95% CI 0.564 - 1.107] |
| **Marital status = Married or partnered now or before** |  | 0.978 |
|  |  | (SE 0.254) |
|  |  | [95% CI 0.588-1.627] |
| **Lives alone = Lives alone** |  | 1.027 |
|  |  | (SE 0.192) |
|  |  | [95% CI 0.712 - 1.480] |
| **Has diabetes = Yes** |  | 1.402** |
|  |  | (SE 0.237) |
|  |  | [95% CI 1.007 - 1.952] |
| **Has cancer = Yes** |  | 2.085* |
|  |  | (SE 0.903) |
|  |  | [95% CI 0.892 - 4.873] |
| **Has osteoporosis = Yes** |  | 0.940 |
|  |  | (SE 0.144) |
|  |  | [95% CI 0.695 - 1.270] |
| **Has a nervous or mental disorder = Yes** |  | 0.987 |
|  |  | (SE 0.174) |
|  |  | [95% CI 0.699-1.394] |
| **Smoke or smoked cigarettes = Yes** |  | 1.660*** |
|  |  | (SE 0.202) |
|  |  | [95% CI 1.308 - 2.107] |
| **Childhood socioeconomic situation = 1, Good** |  | 1.123 |
|  |  | (SE 0.171) |
|  |  | [95% CI 0.834 - 1.512] |
| **Childhood socioeconomic situation= 2, Regular** |  | 1.103 |
|  |  | (SE 0.158) |
|  |  | [95% CI 0.834 - 1.460] |
| **Childhood period where food was scarce and you felt hungry = Yes** |  | 1.374*** |
|  |  | (SE 0.168) |
|  |  | [95% CI 1.082 -1.745] |
| **Insurance type = 2, Private** |  | 0.735 |
|  |  | (SE 0.210) |
|  |  | [95% CI 0.419-1.288] |
| **Insurance type = 3, Public only** |  | 0.893 |
|  |  | (SE 0.105) |
|  |  | [95% CI 0.709 - 1.123] |
| **Constant** | 6.377*** | 4.253*** |
|  | (0.666) | (1.430) |
|  |  |  |
| **Observations** | 3,899 | 3,899 |

Standard errors in parentheses

95% Confidence intervals in brackets

*** p<0.01, ** p<0.05, * p<0.1
